# Semi-Automatic Determination of Rockfall Trajectories

**DOI:** 10.3390/s141018187

**Published:** 2014-09-29

**Authors:** Axel Volkwein, Johannes Klette

**Affiliations:** WSL Swiss Federal Institute for Forest, Snow and Landscape Research, Zürcherstr. 111, Birmensdorf8903, Switzerland; E-Mail: hannes.klette@web.de

**Keywords:** rockfall, trajectory, tracking, measuring

## Abstract

In determining rockfall trajectories in the field, it is essential to calibrate and validate rockfall simulation software. This contribution presents an *in situ* device and a complementary Local Positioning System (LPS) that allow the determination of parts of the trajectory. An assembly of sensors (herein called rockfall sensor) is installed in the falling block recording the 3D accelerations and rotational velocities. The LPS automatically calculates the position of the block along the slope over time based on Wi-Fi signals emitted from the rockfall sensor. The velocity of the block over time is determined through post-processing. The setup of the rockfall sensor is presented followed by proposed calibration and validation procedures. The performance of the LPS is evaluated by means of different experiments. The results allow for a quality analysis of both the obtained field data and the usability of the rockfall sensor for future/further applications in the field.

## 1. Introduction

Good knowledge of rockfall trajectories is essential to calibrate and to validate rockfall simulation software. Modern computational capabilities allow fully featured 3D rockfall simulations considering high resolution digital elevation models, naturally-shaped boulders and diverse ground parameters defining the interaction properties between soils and a falling block, e.g., [1–3]. Hereby, the interaction parameters vary for different ground conditions and have to be pre-set, calibrated and validated. Typical values as shown, e.g., in [1], have been evaluated by comparing simulated rockfalls with traces of real rockfalls in the field and the subsequent analysis of their trajectories [4]. However, the analysis of a single rockfall event usually does not give information about the kind of this event, e.g., whether it was average or extreme. Therefore, the availability of a series of rockfall events enables a statistical analysis of the observed trajectories [5]. Such a series of rockfalls can be produced artificially. 2D or even 3D analysis of the video records and traces on the ground or trees then reconstruct the trajectories [5]. However, this procedure has two main drawbacks. The video analysis of a single trajectory is a very time-consuming process and during the rockfall experiment a clear view of the camera to the block is necessary, as well a good lighting and contrast settings. The analysis of a single video can result in different trajectories depending on the underlying algorithm that converts pixels into a trajectory [6].

The aim, therefore, is to have a system that measures various components of a falling rock's trajectory. Important elements of a trajectory include displacement, velocity and acceleration for both translational and rotational movements, in 3D. Based on this information, further trajectory elements such as kinetic energy, momentum, ground penetration, coefficients of restitution and friction can be calculated or deduced. Table 1 gives an overview on some elements of a trajectory together with possible resolutions and update rates. The proposed resolutions and update rates are rough estimations based on the idea that if these rates are achieved there reconstruction of a trajectory with typical travel velocities of up to 25 m/s is possible. The rates given are governed by the required recording rates stated in the guideline for rockfall protection systems ETAG 027 [7]. Of course, they might vary depending on the local circumstances. For example, the impact time of a boulder onto hard rock is much smaller than a comparable impact on soft ground with correspondingly different accelerations. The single trajectory elements can be retrieved (direct measurement or deduction) through different methods that are also given in Table 1. However, it should be made clear that the different methods result in different resolutions and update rates that do not always meet the proposed ideal ones and additional restriction have to be considered as described in Sections 1.1–1.4.

To detect some of above-mentioned trajectory elements, this contribution proposes and illustrates a prototype of a measurement system within a boulder and its application as previously and briefly announced in [8]. It consists of an assembly of different sensors with a corresponding data logger (herein called rockfall sensor) paired with a Local Positioning System (LPS). It presents a new possibility to detect trajectory elements in the field during real time for a study consisting of numerous samples. The focus, hereby, is on the detection of the position during the rockfall event using the LPS. The measurement is combined with an acceleration and rotation measurement.

The rockfall sensor and the LPS are presented and evaluated in terms of precision, usability and suitability for a systematic and automatic retrieval of trajectories, to facilitate a proper validation of rockfall simulation software.

### 1.1. Common Methods to Track a Rockfall

If a clear view on the rockfall path is available, video recording constitutes one technique of tracking falling blocks. High-speed video cameras, with a frame rate of at least 100 frames per second, provide adequate discretization over time [7]. With an increasing frame rate the high-speed video faces the problem of limited illumination of the scene required for very short shutter opening times. Further, sufficient contrast is required for post-treatment analyses. The achievable precision of the records depends primarily on the boundary conditions at the test site and the resolution of the videos, for example, for a 100-m-long test site, and a frame size of 800 × 600 pxs, the maximum resolution is 8 pxs per meter. Thus, a block with a real diameter of 0.5 m has a pixel size of 4. The absolute position of the boulder for such a setup can be given with high precision that corresponds to a length of 0.3–0.5 m.

A precision of the spatial positions in the field comparable to video records can be obtained by using geodetic instruments, such as tachymeters or differential GPS (DGPS). In this case, rockfall traces on the ground or on plants have to be identified and manually measured after the rockfall event. Additional video records help to clearly identify the impact locations [5]. Thus, the collection of a large number of trajectories, as necessary for many analyses, becomes prohibitively expensive.

Live tracking, using, for example, RADAR (see also Section 1.2), is preferably used to monitor persistent and slow movements in hillslope processes [9–12].

New GPS sensors on the market since 2013 allow an update rate of more than 10 Hz [13], which may be useful for live rockfall tracking usage with a sensor attached to the block. In this case, several GPS antennas have to be distributed over the block to guarantee good GPS signal reception because one single antenna is frequently oriented below the boulder and consequently shaded [13]. Precision of the measurement is expected to be around 1 m, or even better if a differential GPS arrangement can be setup. Furthermore, the test site has to be checked for good GPS reception along the slope when preparing rockfall experiments, especially in steep mountainous areas.

Other techniques of tracking elements in the field employ locally installed systems. Passive radio reflectors have already been used to track sediment particles in rivers using radio-frequency identification techniques (RFID) [14,15]. Magnetic sensors also allow the detection of passing particles [16]. Ergenzinger *et al.* [16] also used active radio transmitters to track sediment transport within rivers. However, all these systems work only for small distances of up to a couple of meters and have only been tested for particle speeds of generally less than 1 m/s. Rough position detection in the field of block impacts on the ground using a wireless geophone network that analyzes the sound travel times was firstly introduced by Cadalbert [17].

If the position (and velocity) of the falling block has to be calculated from the measured 3D accelerations the precise orientation of the boulder must be known, *i.e.*, measured over time. Then, the direction of the block's local accelerations can be transferred to a stationary coordinate system. Some experience with these procedures exists [18,19] but if time integration takes place over a span of several seconds it was shown that, in particular, the position drifts [18]. Therefore, regular checkpoints are necessary where the calculated position can be updated using an additional position measurement during the rockfall event. Then, these position measurements need only a small update rate and the high frequency acceleration measurement allows for a deduction of the trajectory between the known points.

The current absolute vertical position above a reference elevation of the falling block may also be found through a barometric sensor. However, tests with such sensors did not work well under dynamic test conditions because the air pressure changes around the block during its movement [13].

### 1.2. Existing/Known Velocity Measurements

Velocities can be obtained by differentiation of the position data. Fluctuations due to inherent imprecision of the position measurement can be encountered by working with averages over longer time spans or using linear regression models for time periods in which the block moves freely through the air and its trajectory is influenced by gravity only.

Good velocity information can be retrieved when analysing GPS signals. The Doppler effect of the satellites' signals is measurable and these procedures are standard in common GPS devices, such as navigation systems [13].

**Figure 1.**
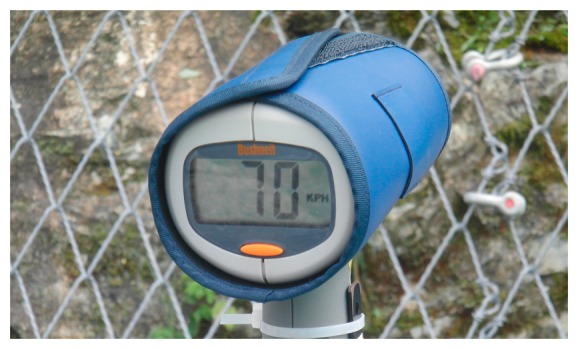
Velocity measurement of a block using RADAR technology.

RADAR measurements are often used to monitor rockfall-prone rock faces (e.g., [9–12]). The measurements provide a 24-h working time both day and night and despite fog or clouds. The achievable resolution goes down to the sub millimeter. Not only detecting changes in the rockface but also to measure the velocity of a falling block (see Figure 1) requires a suitable update rate. In this case, the block must be large enough and maybe be equipped with additional reflectors to guarantee good reflecting signals. Depending on the RADAR device used manual tracking of the block is necessary.

### 1.3. Determination of Accelerations Acting on a Falling Block

Direct measurements of accelerations during rockfall experiments exist for tests of rockfall protection systems with more or less mainly one-dimensional movements [19–23]. The system used in [19] has its origins in testing facilities for dynamic crash tests. In [24,25] acceleration measurements were made for free (non-predicted) movements of test specimen in debris flow or sediment transports.

The acceleration measurement should have a rather sample rate (>1 kHz for dynamic impacts are prescribed in [7], e.g., for force measurements) to map the expected short-timed peaks. The system described in [19] even uses 20 kHz. Measurement ranges of 500 g or even more are used where g is the acceleration due to gravity [19–23].

Double differentiation of position records to obtain accelerations (e.g., video) is not recommended for mainly two reasons: (1) Mostly, the sampling/update rate of the boulders position is lower than the sampling rate necessary to measure short-term peak accelerations (see Table 1);(2) During the impact process the block often experiences the maximum acceleration when the velocity component perpendicular to the ground surface changes from inbound to outbound. This zero-crossing results in only very small corresponding displacements. Therefore, the displacement must be detected with high precision (e.g., millimeters for hard contacts or centimeters for softer ground or protection systems) which is possible only if an impact point is very well known in advance and is filmed close up with high temporal and spatial resolution.

If the velocity of the falling block is measured directly with a suitable resolution and a large sample rate a differentiation of the signal might lead to good acceleration data. However, no experience with such procedures is known so far.

### 1.4. Measurement of Rotational Movements

For the 3D orientation and angular velocities commercially available gyroscope sensors are available that allow for a measurement of these kinetic values sufficient to the needs given in Table 1. Determination of rotations from video records is only recommended if a sufficient visual resolution of the boulder is achieved, *i.e.*, rotation is visible in video. Horizontal orientation also can be measured using a compass-like sensor (shock-resistance required). If boulder accelerations are measured and the sensor resolution is high enough to detect a change of ±g the actual orientation and rotation velocity can be derived.

## 2. System Setup and Evaluation Procedures

**Figure 2.**
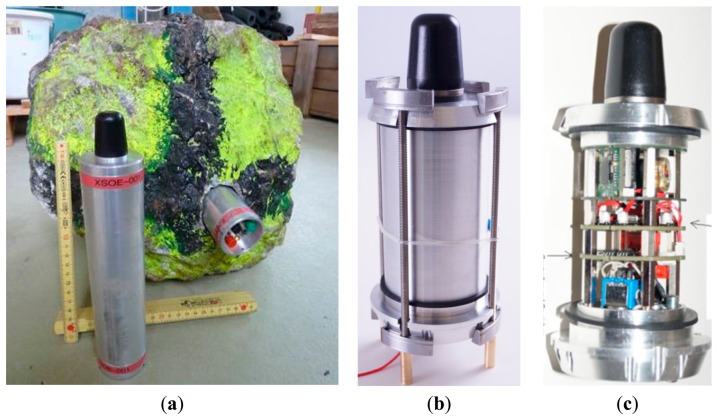
(**a**) Developed rockfall sensor with specially prepared but still natural boulder. The rockfall sensor is fixed fully inside the boulder with wood wedges;(**b**) Rockfall sensor enhanced with fixation clamps;(**c**) Opened rockfall sensor.

Based on the different measurement possibilities shown in the previous section and considering their advantages and disadvantages together with the possibility of combining different measurements in a single device, a special measurement system has been developed. The setup consisting of a unit (see Figure 2) to measure accelerations and rotation rates with high frequency sampling in combination with the LPS is presented in the following section followed by the calibration and validation tests performed. Because of already established acceleration and rotation measurements [19–23] the main focus is on position measurement.

### 2.1. Setup of the Measurement System

Measuring accelerations and rotations is achieved using standard sensors operated by a microcontroller (Atmega2560) with a resolution of 8 bits and a sample rate of about 900 Hz. The aim of the proposed prototype system is to test the general applicability of such a rockfall sensor. Therefore, affordable components with acceptable risk in the case of failure during the field tests were chosen. Better components with, e.g., 16-bit resolution and higher sample rates are recommended (see also Section 4) for operational measurement systems.

The accelerations are measured in three orthogonal directions using a single sensor (Vibration Sensor-Model 832 produced by Measurement Specialties) with a range of ±500 g in each direction. Measurements are carried out piezo-electrically, associated with small sensor drifts over time. The conversion factors from Millivolt per Volt (mV/V) into accelerations have been taken from the factory calibration certificate.

Two different setups were tested to measure the 3D rotational velocities. At first, the 3D rotation sensor L3G4200D (manufacturer: STMicroelectronics) was installed with a measurement range of 2000 °/s. However, initial tests with a 70 kg boulder have shown that higher rotational speeds have to be expected. In a second approach a combination of two 2D sensors LPY4150AL with a measurement range of 6000 °/s (non-amplified) were installed. The sensors only measure rotation rates. The conversion factors of the measured signals from Millivolt per Volt (mV/V) into accelerations have been taken from the factory-based calibration certificate.

**Figure 3.**
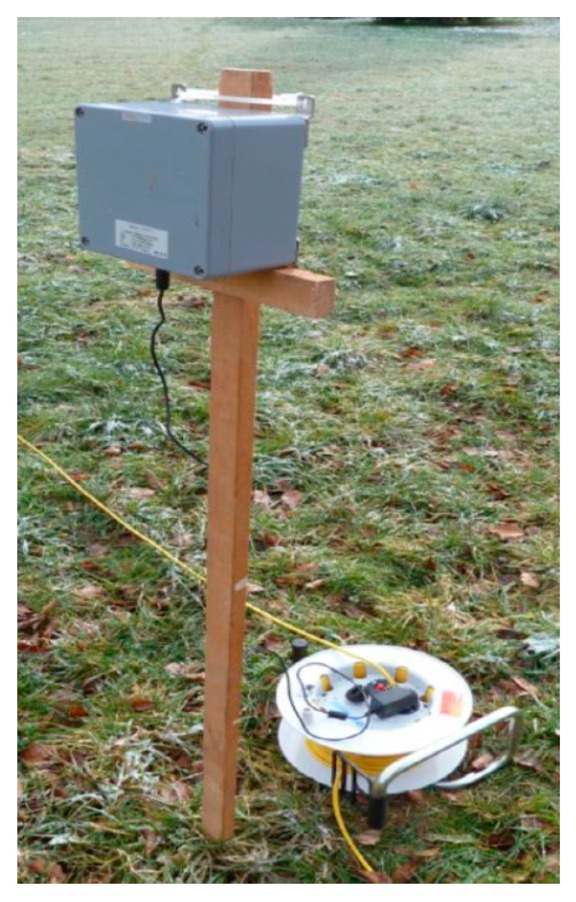
Pseudolite of the local position system in the field with power supply.

The LPS (manufacturer: Nanotron) was installed with a design opposite to that of GPS: within the boulder a Wi-Fi signal is emitted from a single antenna, which is received by at least four so-called pseudolites (see Figure 3) distributed around the testing area. The pseudolites are interconnected wirelessly and linked to a central base station through another Wi-Fi transmitter frequency. The coordinates of the pseudolites in the field must be determined in advance with coordinates coming from GPS or by a survey through a tachymeter, in our case each with a precision of 0.05 m (the influence of different precisions for the pseudolite positions has not been investigated). The position of the boulder is then calculated from the different flight times from emitter to the single pseudolites. The precise algorithm is, however, is proprietary and was not provided by the manufacturer. We assume a differential approach, *i.e.*, the emitter's position is determined by the relative flight time differences rather than on the absolute flight times. The evaluation procedures for the LPS given in Section 2.2.2 are, therefore, set up to understand the performance of the underlying algorithms and the corresponding results. The conclusions can be found in Section 3.4.

When running the LPS software “nanoPAL RTLS Toolbox” the position of the rockfall sensor is visible (live) on the monitor of the base station. The position stored is calculated from an average value in combination with a pre-set constant-velocity prediction. The position is updated at about 15 Hz. The pseudolites have to be positioned along a virtual plane and the actual block position is calculated in relative to this plane. In the momentary configuration, the LPS is limited to 2D by the manufacturer, so only a 2D position with no height information is received.

#### Additional Design Specifications

Measuring rockfalls without interfering with the block is an elegant way of observing natural rockfall events. However, the above listing of possible or necessary measurements shows that measurements directly connected to the falling block are essential. Due to the strong interaction of the boulder with the ground and to avoid modifying the shape of a boulder, measurement systems are ideally placed inside the boulder. Considering the developed rockfall sensor in Figure 2b this means that testing specimen have to be opened up by drilling or similar methods (see Figure 2a). The opening of the boulder does not necessarily have to go through the boulder's center of gravity: The translational acceleration and angular rate are the same for whole boulder and additional acting centrifugal acceleration can be deduced from rotation rates during free flight phases. Fixing the rockfall sensor within the hole was achieved in our case either by the use of wood wedges for the sensor shown in Figure 2a or a specially constructed clamp structure (see Figure 2b). The metallic clamp structure perfectly transmits the boulder accelerations to the corresponding sensor. When wood wedges are used, very strong fixation must be achieved or damping of the signals may occur. Depending on the size of a rock-internal sensor, a fixation of both ends is required, which again needs an accessibility of both sensor ends.

Another important issue is the required shock resistance of the rockfall sensor to an estimated load of at least 500g. That means that a circuit board with a weight of, e.g., 0.05 kg must be able to withstand short duration loads of up to 25 kg. In our case, a certain level of shock resistance has been achieved by the use of only small circuit boards and sufficient bracing elements (Figure 2c). Filling of the rockfall sensor's housing pipe with Epoxy provides further protection but with the loss of the possibility to access the hardware, e.g., for maintenance. The shock resistance condition also influences the data logging and transfer because typical memory cards (e.g., SD/microSD) were found to be insufficiently shock resistant [13]. Therefore, a RAM-based data logging/data storage unit with a post-experiment wire-based data transmission was chosen.

Special focus has to be placed on the triggering of a measurement. Manual triggering is possible if the boulder is accessible immediately before the test (e.g., the red switch on the rockfall sensor visible in the boulder in Figure 2a). Remote triggering based on radio-communication is possible as long as an antenna of the rockfall sensor is well protected during the experiment. Automatic trigger can be realized for free falling blocks if soft-/firmware of the rockfall sensor starts data recording automatically as soon as the vertical acceleration drops by the value of 1g. A flexible solution would be to implement a mechanical triggering using a connection (e.g., a small clamp or magnet) between two electrical contacts. Recording starts as soon as this connection is removed from the rockfall sensor. The latter has been chosen for the version of the rockfall sensor shown in Figure 2b,c.

Recording time and sample rate must fulfil the requirements for various applications such as rock rolling experiments in the field or full-scale tests of rockfall protection systems, *etc.* The rockfall sensor presented here has a data sample rate of about 0.9 kHz. The length of the measurement time had to be chosen in advance. It was set to 2 min, which corresponds to a boulder travelling at 25 m/s along a 3000 m slope because it was estimated to be below this time limit in planned experiments.

Finally, having in mind that a rockfall experiment might damage the instruments, an evaluation of an affordable measurement system is desirable even if that means that, e.g., sensors have to be installed that have some disadvantages, such as a drifting over time of the acceleration sensor.

To measure accelerations and rotations one needs a correct time stamp. This is important because the change of the signals over time can be used to calculate additional values, such as the velocity evolution and actual orientation. For the LPS, extremely precise flight times do not play a significant role as long as the pseudolites are synchronized and the actual position of the boulder is calculated from the relative difference of arrival times. This means that even if the time measurement within the LPS is not absolutely correct, the LPS still calculates the correct position of the rockfall sensor. See also Section 3.4.3 for extended flight times due to a movement of the rockfall sensor out of the virtual plane spanned by the pseudolites.

### 2.2. Calibration + Validation of Measurement Systems

A calibration/validation of such a measurement system is essential. Not only of quality control for the delivered components but mainly as a regular check after a testing series with the test specimen due to the large expected loads as explained above. The validation test for the acceleration sensors can also be used to see whether all components in the rockfall sensor are still rigidly connected to each other.

#### 2.2.1. High Frequency Sampling

The validation of the sample rates requires a time-calibrated reference system with a fixed connection. This could be another data retrieval unit with a verified sample rate and an acceleration sensor. If then both systems are shock-loaded several times the time distance between the single shocks must be identical. Both sample rate and acceleration amplitudes can now be checked. If the shocks of the rockfall sensor are filmed with a synchronized high-speed video camera (frame rate in this case equal to or greater than the sample rate) the video data also allows a verification of the sample rate of the microcontroller of the rockfall sensor.

**Figure 4.**
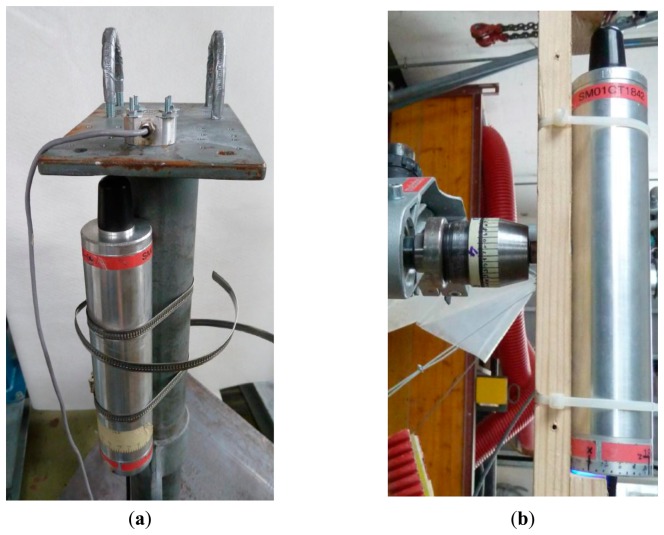
(**a**) Validation test setup for acceleration measurement; (**b**) Validation test setup for angular velocity using a drilling machine.

To check the acceleration measurement in the rockfall sensor a setup with a comparison acceleration measurement system is used. Both systems/sensors were mounted on a small platform that falls downward a few centimeters (see Figure 4) onto a steel surface, damped with some tape to extend the impact time comparable to an impact with the ground in the field. The platform is guided along a steel pipe to confine the movement to the vertical direction. The maximum amplitudes of the rockfall sensor and reference accelerometers are then compared. A reference system with a higher sampling rate (in our case a factor of about 10 was available) can be used to investigate the performance of the rockfall sensor with the given sampling rate. A system with a too-low sampling rate will likely miss the maximum value of short-duration acceleration peaks. If the precise orientation of the acceleration sensor in the rockfall sensor is known, the test can be repeated for all three orthogonal directions. Alternatively, and assuming that the peaks in the individual orthogonal directions happen at the same time, the total acceleration (vector length of single axis accelerations) can be used for calibration purposes with, however, the loss of directional information.

For a check of the angular velocity, a simple setup with a drilling machine is used. The rotational speed of the setup is determined using a high-speed video camera and then compared with the measured angular velocity. This procedure is repeated for all three orthogonal directions. The acceleration sensor also delivers the actual orientation of the rockfall sensor because it measures the inclination with a change of *g* from vertical to horizontal and a change of 2g for an up-down rotation of 180°.

#### 2.2.2. Local Positioning System

**Figure 5.**
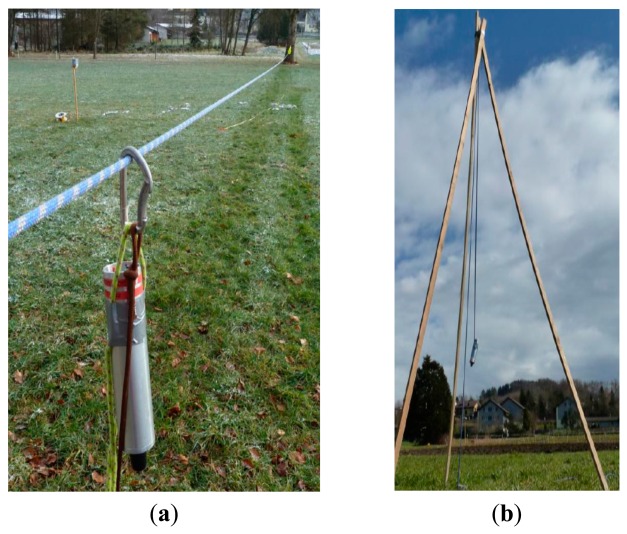
Quasi-static test setup for local positioning system with (**a**) multiple guidance of the rockfall sensor along a spanned rope and (**b**) a study on the influence on the “jump height” of the falling rock.

**Figure 6.**
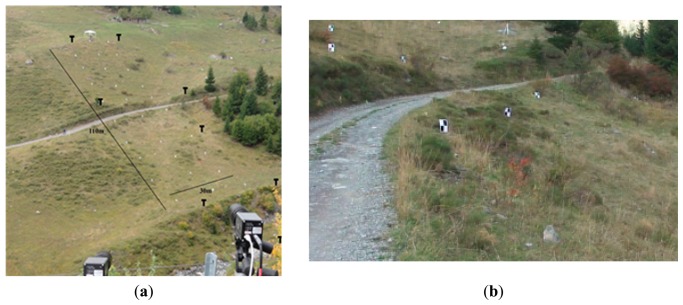
(**a**) Hillslope test site in Vallee de la Sionne VS in Switzerland with positions of pseudolites marked with “T” and (**b**) with a gravel road cutting across the hillslope.

The main focus of the validation process was on the Local Positioning System. Here, what precision can be expected, which conditions the system needs, and where are its limits had to be checked. The first four testing series (a–d) were of a quasi-static type, followed by (e) a dynamic testing setup:

(a) The arrangement of pseudolites has been changed around a ∼10 m× ∼20 m area to identify an influence on the precision as a function of the number of activated pseudolites.
(b) The rockfall sensor was pulled several times along a spanned rope through the ∼10 m × ∼20 m are a (Figure 5a). This setup produces results for both the repeatability of the positions calculated and the maximum deviation from the straight line which results when the rope is projected on to the *x*-*y*-plane of the LPS.
(c) The rockfall sensor was lifted vertically to check the influence of an out-of-plain movement of the 2D position (Figure 5b).
(d) The rockfall sensor was moved within and along the test field and was simultaneously tracked by a tachymeter in auto tracking mode. The comparison of both measurements shows the limits of the position calculation and the precision of the recorded path.
(e) A field test series has been conducted where a 70 kg boulder was released at the top of a slope. The slope was covered with grass and small bushes; there was a gravel road in between, a shadow angle of about 30° and a length of ∼110 m (see Figure 6). A more or less regular grid of black and white targets was distributed along the slope. For the later analysis of the rock rolling experiments, the trajectories were observed using:
  - Local positioning system.
  - High speed video records.
  - Tachymeter with measuring single impact locations and other trajectory points observed from video records.

## 3. Validation/Calibration Results

The setup of the measurement system was tested and evaluated regarding its usability in laboratory and field including the integration into the test boulders, the battery capacity and the triggering mechanism.

### 3.1. Time Measurement/Sampling Rate

A comparison with an independent measurement system, which was set to 10 kHz sampling rate, and parallel high-speed video measurements had an average rockfall sensor sampling rate of 907 Hz in 10 tests. The sampling rate varied between 768 and 1367 Hz.

### 3.2. Acceleration Measurement

The setup shown in Figure 4 allows for checking the acceleration measurements along the different axes and their response orthogonal to the direction of excitation to study the cross sensitivity. Table 2 presents the results of eight excitations along the rockfall sensor's longitudinal axis. To calibrate the rockfall sensor's sensor a linear regression between the reference acceleration (*a_ref_*) and *a_z_* gives a correlation of R^2^=0.64. If $a_{\text{total}}=\sqrt{a_{x}^{2}+a_{y}^{2}+a_{z}^{2}}$ is correlated with *a_ref_* R^2^=0.67 is achieved. The single impacts are distributed over about 80 sample points by the reference sensor and over ∼8 points within the rockfall sensor.

### 3.3. Angular Velocity

**Figure 7.**
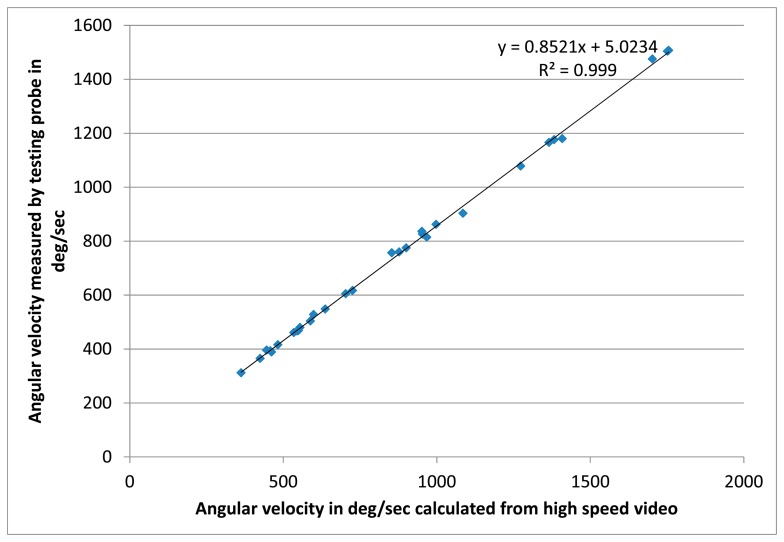
Angular velocity measurements by high-speed video *vs.* angular velocity measurements by testing rockfall sensor with corresponding linear regression.

The rotation sensor was tested for different rotational speeds using the setup shown in Figure 4. The result is shown in Figure 7, where the values measured by the rockfall sensor, are compared to the angular velocities calculated from the analysis of high-speed video records. The corresponding linear regression has R^2^=0.999 (Figure 7).

### 3.4. Local Positioning System

The Wi-Fi-antenna was placed roughly in the center of a natural 70 kg boulder and the Wi-Fi-signal was detected from all pseudolites during all tests independent of the actual orientation of the block. If an artificial boulder made of steel-reinforced concrete is used then the antenna must be positioned close to the surface of the test block because the steel would block the Wi-Fi signal.

On a test field having a size of about 200 m × 50 m with eight pseudolites and a base station all Wi-Fi signals between the pseudolites, between pseudolites and base station and between rockfall sensor and pseudolites enabled a continuous radio communications. Larger test fields might require additional pseudolites, as well as base stations or signal repeating devices, to support the communication between the single pseudolites and the base station. In the ideal case, the radio signals are not be influenced or disturbed by, e.g., nearby buildings to avoid wrong results due to signal reflections.

The pseudolite positions in the field were determined using a tachymeter and a GPS, each with a precision of few centimeters. Electric power for the pseudolites is supplied by batteries. Ideally, their capacity lasts for a full day in the field, even for low temperatures. Alternatively, a combination of electric generator and extension cords along the sides of the test field has been used.

The position detection rate of the LPS varied for the different tests between 14 and 16 Hz, which was visible from the time stamps stored with the calculated positions. The signal-emitting rate of the rockfall sensor is larger (but unknown to us) and the position in the field is filtered over time. The polling rate of the pseudolites by the base station was set to 30 Hz. The software calculates the most probable position by internally predicting the new position, based on the last positions and a fix set velocity prediction. In our case, this preset velocity had a value of 15 m/s for all tests described in Sections 3.4.1–3.4.5.

#### 3.4.1. Linear Repetitive Movements

**Figure 8.**
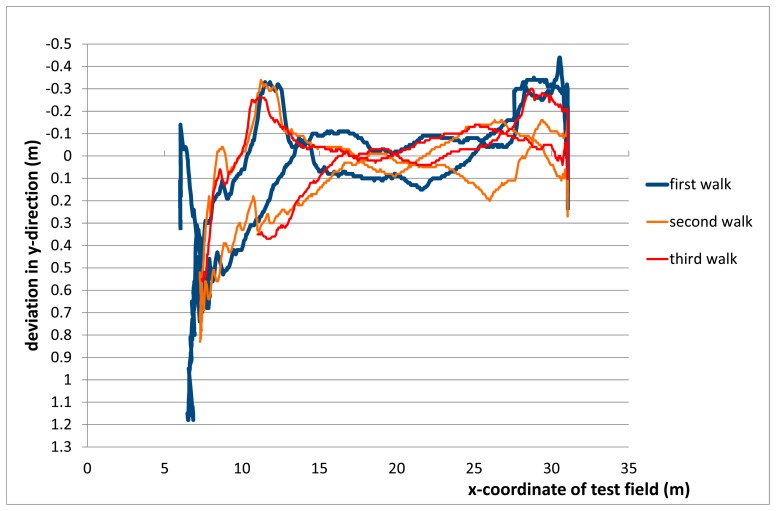
Deviation [m] of detected position [m] in relation to the given straight movement along a spanned rope.

In the first test the rockfall sensor was attached to a stretched 30 m rope inside the pseudolite polygon. The rockfall sensor was then pulled along this rope three times. The deviation of the calculated LPS position to this line is shown in Figure 8. It can be seen that the lateral deviation is around or less than 0.1 m in the center of the pseudolite polygon. At the ends (and closer to the polygon borders) the deviation increases to up to 0.5–1 m. The repeatability of the results for three separate passages is also visible in Figure 8.

#### 3.4.2. Free Movements

**Figure 9.**
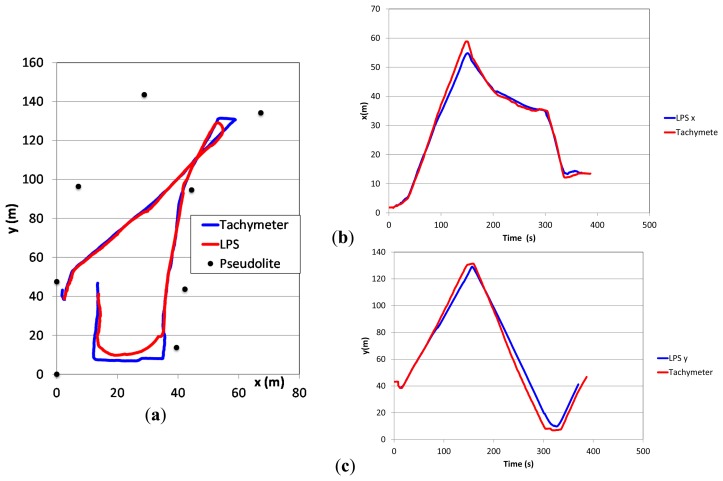
Observed and referenced track on (**a**) a plane field and evolution of (**b**) *x*- and (**c**) *y*-coordinates over time.

When walking freely on a plane field referenced by simultaneous tracking with a tachymeter the reference path and the detected path are shown in Figure 9. As long as the movements happen within the pseudolite polygon the actual position is calculated correctly with the precision described above. Leaving the polygon results in the observed position shown along its edge.

Due to the internal position observation techniques and a filtering over time of several positions sharp track turns are abbreviated and rounded in comparison to the actual movement resulting in an error of up to 5 m. Linear movements are detected with a precision of less than 1 m (see Section 3.4.1).

#### 3.4.3. Influence of “Jump” Height

**Figure 10.**
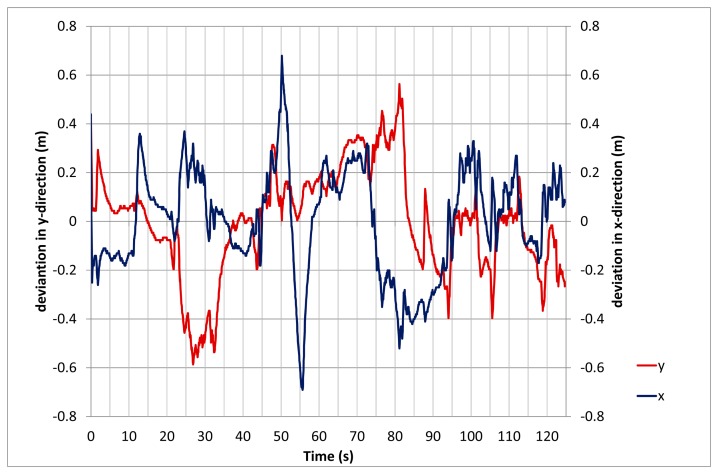
Varying position during changes of heights above ground.

Because the LPS only delivers 2D coordinates, the influence of jump heights within the detected trajectories has to be studied independently. The setup in Figure 5b allows heights, above ground, of up to 4 m. Figure 10 shows the calculated 2D positions of the LPS for the different heights according to Table 3. The graph shows an average error of up to 0.8 m during the height changes but a subsequent improvement once the height of the rockfall sensor remained constant. It is interesting to note that the deviation is reduced during continuous movements of the rockfall sensor, which cannot be explained at the moment, but may be due to the manufacturer's proprietary tracking algorithm. However, this effect is helpful for the application to rockfalls, showing that jumps of the boulder have little influence on the recorded trajectory.

#### 3.4.4. Number and Positions of Pseudolites

**Figure 11.**
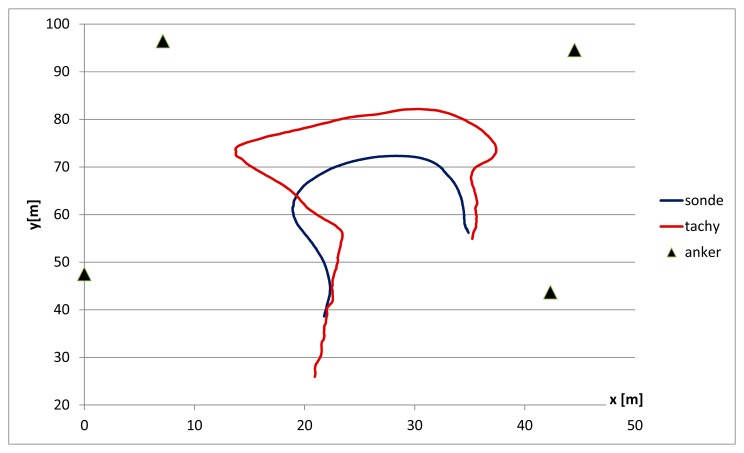
Comparison between LPS and tachymeter measurement with only four active pseudolites.

Changing the number of pseudolites and varying their position in the field showed that the calculated LPS positions improve with an increasing number of pseudolites. If the number of pseudolites is reduced the above described effects of abbreviated turns increase (Figure 11). With a stationary position the same coordinates were observed. In total, by the use of fewer sensors the systems seems to need more time to calculate a correct position.

The distance between neighbouring pseudolites should not exceed 50m as recommended by the manufacturer of the LPS.

#### 3.4.5. Rock Rolling Experiments

**Figure 12.**
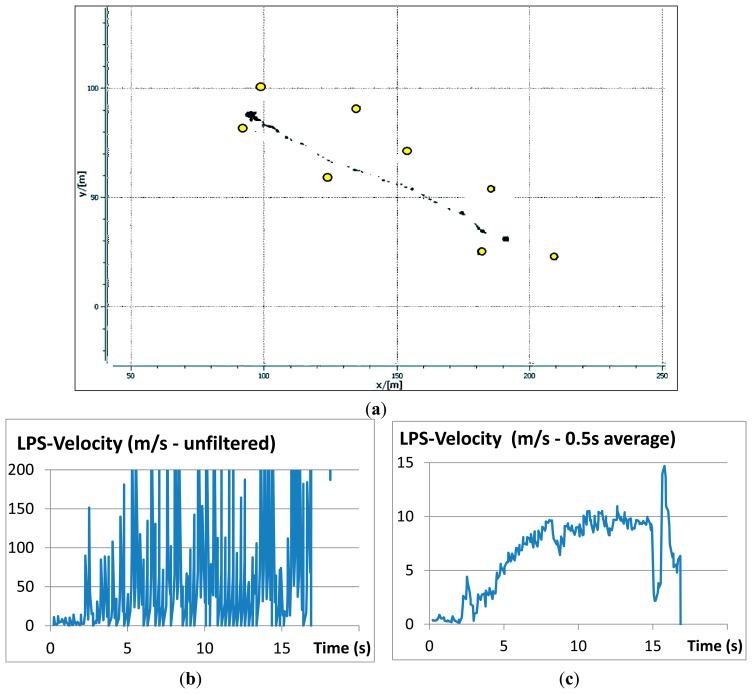
(**a**) LPS records during field test including pseudolites positions as provided by the software at the LPS base station; (**b**) Unfiltered velocity computed from LPS data; (**c**) computed velocity data with 0.5 s running average.

Figure 12 shows the results of a LPS-measured trajectory with (a) the real-time output on the monitor of the base station which is identical to the trajectory saved to disc and (b, c) the evolution of the velocity over time derived from trajectory data. The cessation of motion was due to the boulder coming to a sudden rest in a riverbed at the base of the slope.

**Figure 13.**
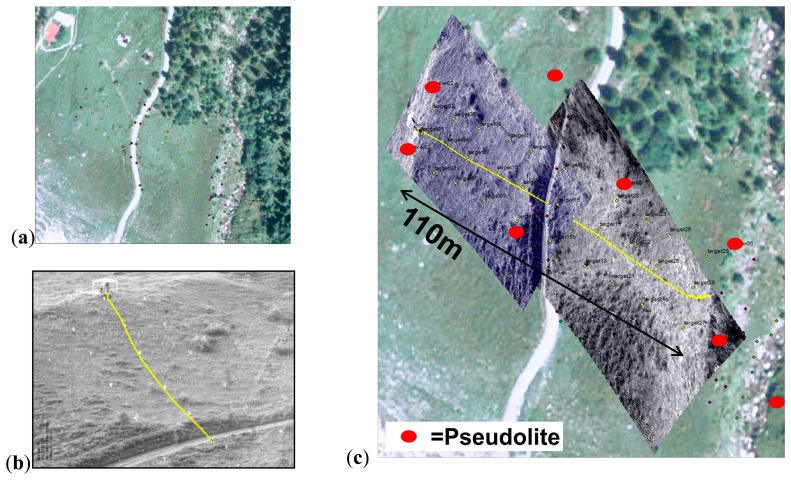
Combination of (**a**) surveyed field targets and (**b**) high speed video image into (**c**) GIS.

**Figure 14.**
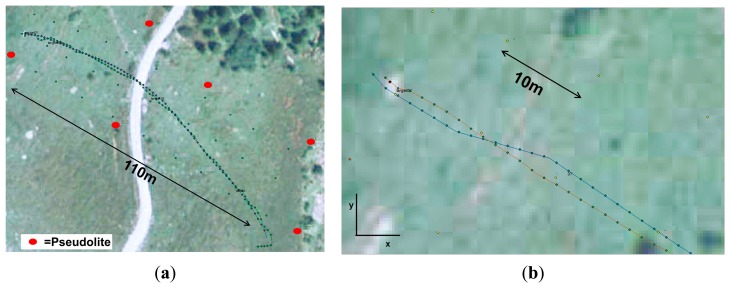
Overlay of video trajectory and LPS trajectory: (**a**) full length; (**b**) detail with regularly created points along the trajectory lines.

For the analyses of the video records the software Kinovea was used. The trajectories were first measured automatically and then corrected manually, where necessary, resulting in a trajectory plotted on the camera image (Figure 13b). Because the focus was on the movements along the ground, jumps of the falling block were corrected manually to a straight line between beginning and end of a jump. The resulting image is geo-referenced (Figure 13c) using the surveyed targets in the field (Figure 13a). In the same way, the LPS trajectory was imported into a GIS enabling a superposition as shown in Figure 14a. Such a graphical procedure produced better results compared to a superposition of the *x*-*y*-coordinates calculated from the video records and LPS records separately. For a quantitative comparison of the trajectories virtual points have been distributed regularly along the single trajectories (Figure 14b). The seven measured LPS trajectories fit the corresponding video trajectories with average deviations of 0.11–1.01 m (see Table 4).

**Figure 15.**
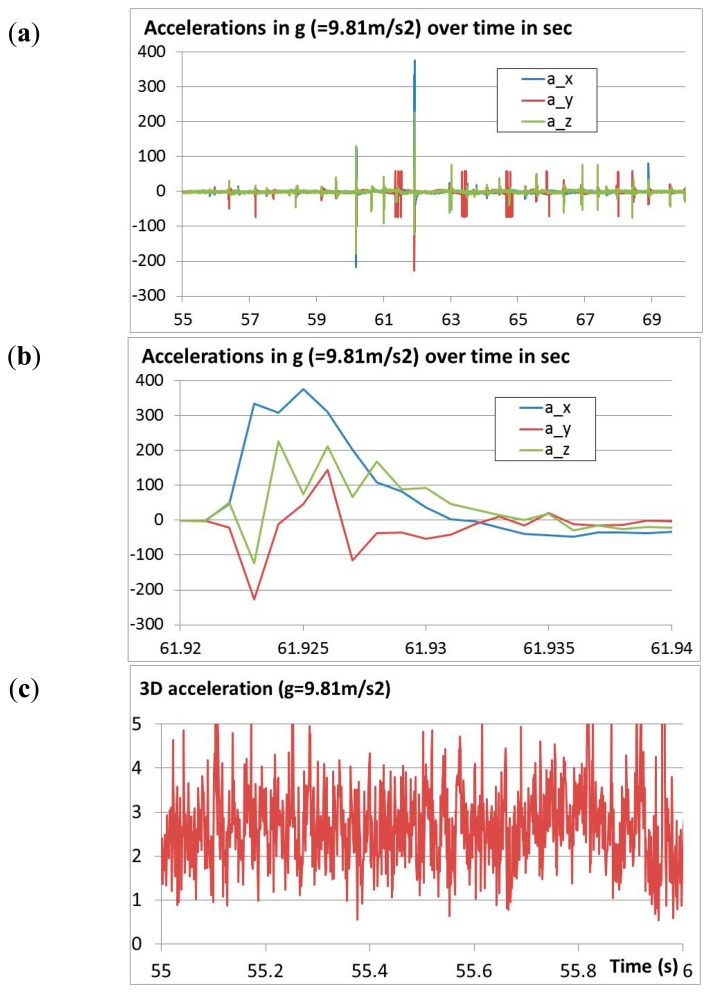
Field test acceleration measurement during (**a**) full time span; (**b**) hard impact and (**c**) state of rest immediately before the test.

During the field tests also accelerations and rotations were measured at a rate of about 900 Hz. The accelerations are shown in Figure 15a. Usually, the accelerations remained below 100g. Single impacts reached almost 400g (Figure 15b). The acceleration sensor showed a noise level of about ±2g (Figure 15c). However, this noise shows some remarkable peaks that follow a certain rhythm of about 30 Hz.

**Figure 16.**
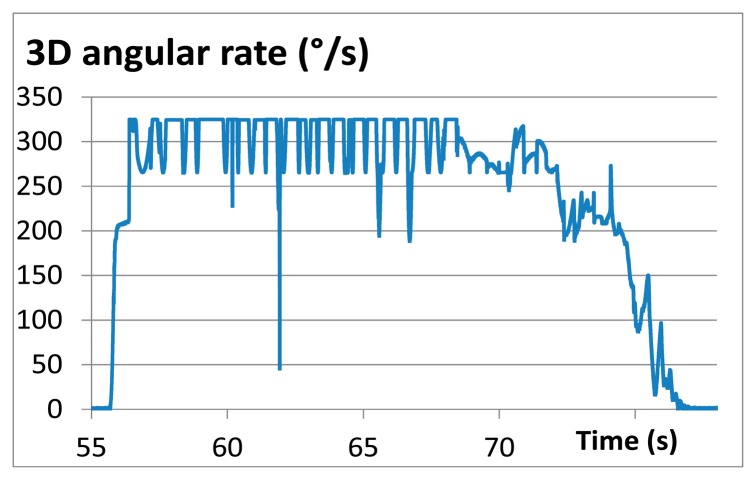
Field test rotation measurement with limited measurement range.

The rotational velocities were not completely measured, however, because the internal measurement firmware was set by accident to a maximum angular rate of 320 degrees per second. The angular rate shown in Figure 16 is, therefore, limited at this value and the maximum rotational velocities were not obtained during the field test.

## 4. Evaluation and Discussion

The prototype of the rockfall trajectory measurement system consisting of a rockfalls sensor combined with an LPS was tested regarding its suitability for calibration of the sensors and its applicability in the field. It was found that the setup is usable in the field but has its limitations. In the following, some properties are summarized that are important for the later application (e.g., high frequency sample rate, size of test field), drawbacks are listed and potential points for improvement are suggested.

It was possible to measure a reasonable space-time trajectory of the boulder. Battery capacity of the rockfall sensor is sufficient when charging the battery during pauses between the single experiments. During our tests the rockfall sensor was not damaged in any of the impacts. Changing the data storage inside the boulder from RAM to SSD enables post-event data retrieval, even if the battery power drops beforehand.

A thorough check of a new measurement system is essential prior to use. In our case, for example, we discovered that the varying sample rate of the internal microprocessor is a major issue. To address this topic, the actual timestamp has to be stored with the data and the sample rate cannot be assumed as constant during post-processing.

The acceleration data provides information on the intensity of single impacts. This information can be directly used to calibrate the ground behaviour with trajectory simulation software. The actual sampling rate of about 900 Hz seems to resolve the impacts on the ground in sufficient detail for elongated impact peaks. If hard impacts (e.g., contact with large solid rocks or bedrock) are expected, then a higher sampling rate might be necessary. It is difficult to predict the necessary minimum sampling rate in advance without additional systematic testing. However, when looking at acceleration data during post-analysis and an acceleration curve can be drawn smoothly based on the measured sampling points then the sampling rate is assumed to be sufficient for the expected applications. This point also has to be considered when calibrating the sensor. The reference acceleration must last long enough for it to be received with the actual sampling rate, *i.e.*, if a signal peak consists of only a few sample values a precise statement on the correctness of the peak magnitude is not possible. The calibration scheme, as described in Section 2.2.1, should be modified to achieve softer impact because it was observed that the duration of the single shocks during impact of the rockfall sensor on the ground exhibits characteristics of being still too hard even if damped using tape. Further, the impact from the calibration setup did not map the impacts we typically observed later in the field.

The transverse sensitivity of the accelerometer is too large. This means that if the sensor is accelerated in only one direction the two other orthogonal directions also deliver signals for accelerations that do not exist in reality. The observed transverse sensitivity of up to 20% even exceeds that given in the sensors data sheet (<10%). The reason behind this could not be fully solved. Geometrically correct installation of the experiment was verified, as well as the calibration of the reference sensor. Perhaps, the structure of the rockfall sensor with screw fixation along the longitudinal axis (see Figure 2c) allows an unwanted lateral movement of the circuit board containing the acceleration sensor. Due to this high transverse sensitivity, the total acceleration, being the length of the acceleration vector, also does not result in plausible total impact reaction magnitudes. Finally, the piezoelectric acceleration sensor generated a non-negligible amount of noise. Since the noise still has some remarkably clear and regular peaks, it also might be induced by another electronic component of the rockfall sensor and the LPS sending unit. Because, for the moment the pseudolites are polled with a frequency of 30 Hz, it is assumed that the LPS sending unit might emit its signals with a similar frequency. A deduction of the actual orientation of the rockfall sensor based on acceleration data under pure rotation was not possible. A change to a better capacitive or piezo-resistive acceleration sensor or even a combined rotation speed/accelerations sensor such as IES 3106 by the manufacturer Humanetics, as used successfully in [20], would be better.

The same also can be recommended for the data logger. The low-price solution with just an 8-bit resolution can be improved without the risk of losing the rockfall sensor during an experiment. With the experience and confidence in the robustness of the rockfall sensor already obtained from the experiments performed, if would now be reasonable to invest in a more expensive equipment as reasonable because a loss of the sensor can be more or less ruled out.

The LPS delivers position information on a roughly 200 m × 50 m wide field and with eight pseudolites at 14–16 Hz. The reason for the variation of the detection rate is not clear yet. We assume temporary signal losses during block movement and/or an influence of the task manager of the base station computer. The precision compared to proper video records was found to be reasonably good even if not meeting the requirements of Table 1. Results improve with increasing numbers of pseudolites and it is desirable to keep the distance between them below 50 m. Sharp turns are rounded-off by the underlying algorithms. However, this fact is considered to be irrelevant in the field of rockfalls because sharp turns usually do not occur. A full study on the sensitivity regarding turning radius and turning angle has not been performed yet. For slightly curved trajectories the LPS provides reasonable results at a resolution comparable to video analysis. The time synchronization with a video trajectory, or even with accelerometer data, is difficult due to internal filtering in the underlying algorithms of the LPS. If an LPS could also deliver Doppler effect data the velocity could be determined [13].

The LPS in its actual configuration delivers the positions in 2D coordinates only. An extension to 3D seems possible if the underlying algorithms are extended, but, then, some of the pseudolites would have to be positioned at different heights above the ground to improve tracking of the movement in three dimensions. This necessitates further research to determine whether the precision of the LPS is sufficient to resolve the jump heights correctly. With the present configuration a proper positioning can take place as long as the slope has a fairly constant inclination and the pseudolites are arranged within an approximate plane. Small jump heights do not significantly influence the trajectory. The underlying LPS algorithm considers the runtime measurements of the single pseudolites relative to each other and not to an absolute datum. The latter is necessary as part of the efforts to reconstruct 3D positions with no additional pseudolites at different heights above the ground.

## 5. Conclusions

A new system has been introduced, which helps to detect the trajectories of artificial rockfalls in the field. It is proposed as a first step towards an automatic retrieval of trajectories. The rockfall sensor contains typical sensor technologies for the detection of impacts and rotation speed. The innovation of the rockfall sensor lies in the implementation of a local positioning system (LPS). The system analyses Wi-Fi signals regarding their different runtimes to some so-called pseudolites (receivers) distributed in a polygon around the test site. Wave-form information of the signals is not yet available from the manufacturer. If available, velocity information based on Doppler analyses will be possible as it is already common practice when analysing GPS signals.

The existing rockfall sensor is directly usable forfield-testing. Compared to traditional video records and their analysis a number of trajectories—required for statistical analysis [26,27]—can be performed and analyzed avoiding some disadvantages of video analysis, such as correct lighting, good contrast of scenery and falling rocks, sufficient image resolution, irregular topography, continuous visibility of the block regarding shadowing effects or flora, and the number of cameras necessary to track a single trajectory.

As the video records usually have higher precision randomly performed additional video records are still useful because the precision achievable from video still might be better compared from LPS and the geometric conditions as described in Section 1.1.

The system described herein is a prototype. Some improvements can be considered for future sensors for larger research projects or other applications. For the detection of accelerations and rotations a large number of sensors and data loggers are available. A more stable accelerometer and rotation sensor paired with a data logger with high (e.g., 20 kHz) and more constant sampling rates might enable a 3D reconstruction of a trajectory through integration of the data over time in combination, with some reference points along the slope obtained through LPS, video or geodetic measurements, thereby avoiding typical drifts due to the integration. Additionally, the LPS can be improved by an extension to 3D. Alternatively, the handling of GPS sensors with an update rate of up to 18 Hz and a precision of 1 m can be tested for their suitability in the field. However, perhaps other indoor location systems could be evaluated for their usefulness. However, the main condition is and will be an adequate shock resistance regarding all electronic components built into the boulder.

## Figures and Tables

**Table 1.** Elements of rockfall trajectory with expected range, proposed measurement precision and possible retrieval techniques. The given ranges are estimates because these values might vary strongly depending of the boundary conditions of a rockfall event.

**3D Variable**	Expected Range *	Proposed Ideal Precision *	Proposed Minimum to Ideal Update Rate *	Theoretical Data Deduction Methods **
Position	Slope area	0.2–1.0 m	10–100 Hz	Direct measurement Video Barometer → Height GPS 2D/3D LPS 2D/3D Double integration of acceleration Integration of velocity

Velocity	0–25 m/s	0.5–1 m/s	10–100 Hz	Direct measurement RADAR (1D) LASER (1D) Doppler effects of radio signals Integration of acceleration Differentiation of position

Acceleration	0–500 g	0.5 g	1–10 kHz	Direct measurement Double differentiation of position Differentiation of velocity

Orientation	0°–360°	10°	10–100 Hz	Direct measurement Differentiation of angular velocity Deducible from accelerations

Angular velocity	2000–6000°/s	90°/s	10–100 Hz	Direct measurement Integration of angular velocity Deducible from accelerations

* The ranges given might vary depending on conditions of the rockfall site;

** The different data deduction methods might result in different update rates and precisions achievable.

**Table 2.** Acceleration of reference sensor *a_ref_* compared to measured excitation of rockfall sensor in the longitudinal (*x*) and lateral (*y*,*z*) directions. *a_total_* is the vector sum of *a_x_*, *a_y_*,*a_z_*.

**Test**	***a****_ref_* **[g]**	***a****_x_* **[g]**	***a****_y_* **[g]**	***a****_z_* **[g]**	***a****_total_* **[g]**
1	49.371	49.376	17.374	6.281	52.719
2	67.571	103.194	20.527	10.607	105.749
3	73.788	93.51	17.83	6.172	95.395
4	90.635	99.194	16.163	39.015	107.81
5	97.128	150.774	14.078	33.747	155.145
6	132.393	186.087	17.228	14.61	187.453
7	137.284	119.281	24.312	22.706	123.832
8	155.601	157.605	38.128	41.117	167.283

**Table 3.** Height program of the LPS over time. The 5-s-intervals omitted in the table describe the movement to the next height.

**Time [s]**	**Height above Ground [m]**
5–10	1.4 (height of pseudolites)
15–20	2
25–30	3
35–40	4
45–50	0
55–60	1
65–70	2
75–80	3
85–90	2
90–125	0–4 m (varying)

**Table 4.** Deviation of LPS trajectory from video trajectory.

**Trajectory No.**	**Average Deviation [m]**	**Standard Deviation**	**Variance**
1	0.474	0.675	0.456
2	−0.112	0.769	0.591
3	−0.199	0.516	0.262
4	0.519	1.007	1.01
5	0.646	0.526	0.276
6	−0.237	0.612	0.374
7	1.009	1.041	1.084
